# Hepatic Sarcoidosis Found Incidentally in a Patient Presenting With Recurrent Clostridium difficile Infections

**DOI:** 10.7759/cureus.55316

**Published:** 2024-03-01

**Authors:** Richard Mitchell, Haidar Khan, Jonathan Vincent M Reyes

**Affiliations:** 1 Internal Medicine, Icahn School of Medicine at Mount Sinai/NYC Health + Hospitals-Elmhurst, Queens, USA

**Keywords:** sarcoidosis, extrapulmonary manifestations of sarcoidosis, hepatic sarcoidosis, extrapulmonary sarcoidosis, hepatology

## Abstract

A 50-year-old female who presented to our hospital for recurrent diarrhea was found to have worsening aminotransferase and alkaline phosphatase levels. Workup revealed lymphadenopathy and hepatomegaly prompting a biopsy of the liver and axillary lymph node, confirming a diagnosis of hepatic sarcoidosis. Our patient later developed cutaneous sarcoidosis. She is currently asymptomatic and is followed by gastroenterology, pulmonary, and dermatology. Recognition of extrapulmonary manifestations of sarcoidosis is important for proper management of patients. Treatment often requires a multidisciplinary approach when more than one organ system is involved.

## Introduction

Sarcoidosis is an autoimmune disorder that can affect multiple organ systems. The most commonly impacted organ system is the lung, affecting over 90% of patients. Hepatic involvement can be found in up to 65% of those with sarcoidosis [1]. Sarcoidosis exhibits a spectrum of clinical presentations when it comes to liver involvement. This spectrum spans from asymptomatic cases with mild elevation in liver enzymes [2] to manifestations such as organomegaly, hepatitis, and, in, more severe instances, cirrhosis [3].

Recognition of extrapulmonary manifestations of sarcoidosis is necessary to initiate proper management. This case report describes a woman presenting with nonspecific symptoms who was found to have hepatic sarcoidosis (HS). The patient underwent extensive workup and, ultimately, needed a liver biopsy to confirm the diagnosis. This case highlights the difficulties in diagnosing HS and the importance of a thorough workup.

## Case presentation

A Bengali female in her early 50s with a past medical history of diabetes mellitus, hypothyroidism secondary to total thyroidectomy, and prior *Clostridium difficile* infection initially presented to our hospital for recurrent *C. difficile* infection for the third time. On presentation, she had a mild elevation in her aminotransferases and alkaline phosphatase. During her hospital course, she developed worsening abdominal pain with dramatic elevation of aspartate transaminase to 303 U/L, alanine transaminase to 125 U/L, and alkaline phosphatase to 607 U/L.

**Figure 1:**
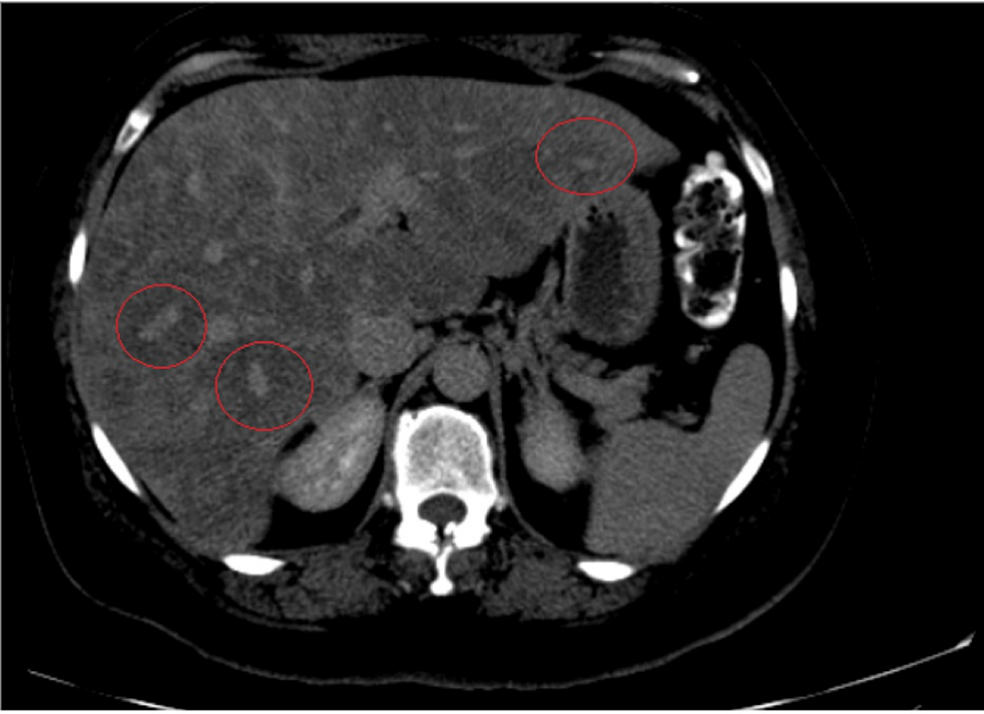
Hepatic hypodensities. CT of the abdomen and pelvis with contrast showing decreased attenuation with diffuse hypodensities (red circles), suggesting spared areas but no clear nodules in the liver.

**Figure 2:**
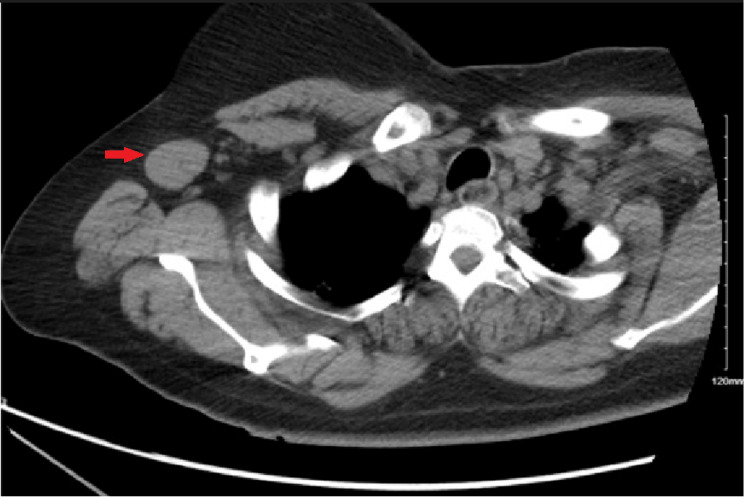
Right axillary lymph node. CT of the chest showing a right axillary lymph node (highlighted by the red arrow).

It was thought that her elevated liver enzymes were secondary to fluconazole, taken for suspected cutaneous candida, and/or cefepime, taken to cover intra-abdominal infections. However, liver enzymes failed to improve after the removal of the suspected offending agents. Laboratory workup for elevated liver enzymes such as autoimmune hepatitis and viral hepatitis was negative. Given the lack of improvement of aminotransferases, abdominal imaging was pursued. Abdominal ultrasound showed hepatomegaly and significant fatty liver infiltration, and CT of the abdomen and pelvis revealed decreased attenuation and diffuse hypodensities with no clear nodules within the liver (Figure 1). There was some concern for metastatic liver disease, so CT of the chest was pursued which showed granulomatous lung disease with mesenteric and hilar adenopathy. CT of the chest also showed a right axillary node and a left axillary node (Figure 2). Given these findings, there were additional concerns for lymphoma, but it was discovered that the patient had a lymph node biopsy done at an outside hospital which was positive for sarcoidosis. Therefore, hepatic involvement of sarcoidosis was the more favorable diagnosis.

To confirm hepatic sarcoidosis and rule out other pathologies, the patient underwent an interventional radiology CT-guided biopsy of the right hepatic lobe, left hepatic lobe, and an enlarged right axillary lymph node. Biopsy for the axillary lymph node revealed multiple non-necrotizing epithelioid granulomas, some of which appeared confluent. No mycobacteria or fungi were detected by acid-fast bacilli and periodic acid-Schiff/Grocott methenamine silver stains. The liver biopsies showed noncaseating granulomas, ductular reactions, and mild mixed inflammation in the portal tracts. The findings of the biopsies were consistent with a diagnosis of pulmonary and hepatic sarcoidosis. Screening for ocular sarcoidosis was negative. She was discharged after improvement of her abdominal pain.

The discharge plan was to follow up with the pulmonary sarcoid clinic and gastroenterology. Oral prednisone was started a couple of months later when a progression of her pulmonary symptoms was noted. Prednisone had to be stopped because of uncontrolled blood glucose, and hydroxychloroquine was started. She was referred to dermatology for newly developed skin lesions on the scalp and left arm. She was treated, empirically, with topical steroids for likely cutaneous sarcoidosis.

She has had no hospital admissions related to her sarcoidosis for the past five years. The patient continues to follow up with pulmonology, dermatology, and gastroenterology for long-term management of sarcoidosis. On her last pulmonary visit, she was noted to have nearly normal and stable pulmonary function. Aminotransferases and alkaline phosphatase are still mildly elevated, but she denies any further abdominal pain. The patient continues to take hydroxychloroquine, and her cutaneous symptoms of sarcoidosis have been controlled with triamcinolone ointment.

## Discussion

The prevalence of sarcoidosis has been estimated to be 40 per 100,000, with 80,000 new cases per year [4]. Hepatic involvement can be found in up to 65% of those with sarcoidosis [1]. HS is typically seen in the setting of underlying pulmonary involvement as isolated HS is rare. In one study, up to 84% of patients with HS had pulmonary involvement, while 15% had none [2]. Another study reported that 23% of patients had isolated liver disease [5]. African American individuals appear to be most affected by sarcoidosis, with three times higher incidence rates [4]. Females are affected more than males. However, one study showed that race, sex, or age did not appear to have a risk of hepatic involvement in individuals with sarcoidosis [1]. However, the study was limited due to its small population size.

The presentation of HS varies. Some studies showed that patients are asymptomatic [2] while others reported patients presenting with symptoms such as fatigue, pruritus, and weight loss [1]. Our patient presented asymptomatically, and workup was pursued secondary to elevated liver enzymes. Rarely does HS present as cirrhosis on diagnosis. A retrospective population-based study from 1976 to 2013 found only two patients presenting with cirrhosis on diagnosis [2]. Other presentations of HS have been reported. Gaduputi et al. reported a case in which HS presented as cholangitis secondary to biliary obstruction from sarcoid granulomas [6]. HS can also be mistaken for hepatic metastases [7,8]. The majority of patients are found to have elevated alkaline phosphatase and/or gamma-glutamyl transferase, resembling a cholestatic pattern of liver injury (1,2). This was consistent with the elevated alkaline phosphatase present in our patient on hospital admission. The role of obtaining angiotensinogen-converting enzyme (ACE) is controversial. ACE level has low positive and negative predictive values in sarcoidosis. Additionally, ACE has poor sensitivity with HS, as with systemic sarcoidosis [2], but may be supportive if elevated [1]. Imaging is generally nonspecific for HS. CT of the liver most commonly shows hepatomegaly with a homogenous appearance [9]. Focal nodules can be visualized in some patients with HS but this is not as common. A few cases report HS showing multiple small nodular lesions [8] or large (4 cm) nodular formation [10]. A liver biopsy, for histopathological examination, is the gold standard if the diagnosis is uncertain. Additionally, biopsy can also be pursued when aminotransferase levels are at least two-fold above the upper limit of normal [11]. Liver biopsy was pursued in our patient given the uncertainty of diagnosis. Histopathology shows noncaseating granulomas with macrophages and rings of surrounding inflammatory cells. Staining with acid-fast bacteria should be obtained to rule out mycobacteria.

HS needs to be differentiated from other liver diseases, especially other granulomatous diseases. Sarcoid may account for 12%-30% of granulomatous liver disease [12]. Other causes of hepatic granulomatous disease include tuberculous, primary biliary cirrhosis, drugs such as allopurinol and sulfonamides, and lymphomas. Furthermore, HS has been reported to resemble cholangiocellular carcinoma [13] and primary sclerosing cholangitis [14]. Additionally, it is important to rule out malignant causes of jaundice or liver injury. Gupta et al. reported a case of a patient with known HS who developed a recurrence of jaundice and was found to have pancreatic adenocarcinoma [15].

Treatment of HS is usually warranted when there is elevation of aminotransferase or other evidence of hepatic injury. Oral glucocorticoids are the most common treatment. Other medications include anti-metabolites and biologic agents. The use of steroids has been shown to reduce aminotransferase elevation back to normal or close to normal [1,2]. Our patient was started on oral steroids once pulmonary symptoms developed. Treatment in our patient was particularly challenging because of persistently elevated blood glucose levels while on oral prednisone. This required keeping her on hydroxychloroquine only. However, while on oral prednisone, our patient’s liver enzymes returned to normal limits. Once the steroid taper was started, there was an elevation of liver enzymes to slightly above normal limits which has remained elevated. Some studies suggest ursodeoxycholic acid as it was shown to be beneficial in patients suffering from pruritus [9]. However, this study had a small sample size, and further studies are needed for validation. Surveillance of HS has not been standardized which makes management and follow-up challenging.

Cirrhosis is a rare complication of HS. Some retrospective studies have reported cirrhosis in four [2], and seven [1] individuals with HS. Ghoneim et al. described two cases of HS leading to cirrhosis in the setting of alcohol use [3]. An even rare complication of HS is hepatopulmonary syndrome (HPS). It has only been reported in two cases in literature [16,17]. In these two cases, HPS was a result of cirrhosis caused by HS. HS has also been associated with an increased risk of hepatocellular carcinoma [18] and Budd-Chiari syndrome [19].

## Conclusions

Sarcoidosis is a relatively common autoimmune disorder and knowledge of hepatic involvement will prompt earlier monitoring and management of these patients. Management can be particularly challenging if undesirable side effects arise.
